# Clinical outcomes of pre-loaded ultra-thin DSAEK and pre-loaded DMEK

**DOI:** 10.1136/bmjophth-2020-000546

**Published:** 2020-10-16

**Authors:** Vito Romano, Luca Pagano, Kunal A Gadhvi, Giulia Coco, Mitchell Titley, Matthew Thomas Fenech, Stefano Ferrari, Hannah J Levis, Mohit Parekh, Stephen Kaye

**Affiliations:** 1 Ophthalmology, Royal Liverpool University Hospital, Liverpool, UK; 2 Institute of Life Course and Medical Sciences, University of Liverpool, Liverpool, UK; 3 Instituto Universitario Fernandez-Vega, Universidad de Oviedo and Fundacion de Investigacion Oftalmologica, Oviedo, Spain; 4 Ophthalmology, Humanitas Research Hospital, Milan, Italy; 5 Ophthalmology, Universita degli Studi di Roma Tor Vergata, Roma, Italy; 6 Venice Eye Bank, Venice, Italy; 7 Institute of Ophthalmology, University College London, London, UK

**Keywords:** cornea

## Abstract

**Objective:**

To compare clinical outcomes and complications between pre-loaded ultra-thin Descemet stripping automated endothelialkeratoplasty (pl-UT-DSAEK) and pre-loaded Descemet membrane endothelial keratoplasty (pl-DMEK).

**Methods and analysis:**

Comparative study in patients with endothelial dysfunction associated with Fuchs endothelial corneal dystrophy and pseudophakic bullous keratopathy who underwent pl-UT-DSAEK or pl-DMEK transplants. For both groups, the tissues were pre-loaded at the Fondazione Banca degli Occhi del Veneto (Venice, Italy) and shipped to The Royal Liverpool University Hospital (Liverpool, UK). Best corrected visual acuity (BCVA) and re-bubbling rates were the main outcome measures.

**Results:**

56 eyes of 56 patients were included. 31 received pl-UT-DSAEK and 25 received pl-DMEK. At 12 months, BCVA (LogMAR) was significantly better for pl-DMEK (0.17±0.20 LogMAR) compared with pl-UT-DSAEK (0.37±0.37 LogMAR, p<0.01). The percentage of people that achieved ≥20/30 was significantly higher in the pl-DMEK group. The rate of re-bubbling, however, was significantly higher for pl-DMEK (44.0%) than for Pl-UT-DSAEK (12.9%), p<0.01.

**Conclusion:**

Pl-DMEK offers better BCVA than pl-UT-DSAEK. The higher re-bubbling rate associated with pre-loaded DMEK is of concern.

**Key messages**

**What is already known about this subject?**

- Surgeon prepared Descemet membrane endothelial keratoplasty (DMEK) offers better visual acuity than Descemet stripping automated endothelialkeratoplasty (DSAEK).

**What are the new findings?**

- Pre-loaded DMEK offers better visual acuity than ultra-thin DSAEK (UT-DSAEK) at 1-year follow-up.
- Pre-loaded DMEK has a higher re-bubbling rate compared with pre-loaded UT-DSAEK.

**How might these results change the focus of research or clinical practice?**

- Eye bank prepared tissues have greater consistency, provide validated tissue and present good clinical outcomes despite the high re-bubbling rate.

## Introduction

Endothelial keratoplasty (EK) is a selective corneal surgical technique that replaces the diseased endothelial layer with a healthy donor endothelium. It is a minimally invasive procedure and therefore it has several advantages such as a fast rehabilitation rate and better visual outcomes compared with full thickness keratoplasty.^1–3^ With the evolution of techniques in EK such as Descemet stripping automated endothelial keratoplasty (DSAEK), ultra-thin DSAEK (UT-DSAEK) and Descemet membrane endothelial keratoplasty (DMEK), there was a steepening of the learning curve and complication rates.^4–7^ Advances in the field of eye banking has resulted in preparation and validation of tissues suitable for such selective procedures.^8^ Recently, Dunker *et al*, compared UT-DSAEK and DMEK in a randomised clinical trial and concluded that they did not differ in visual outcome at 12 months, even if the percentage of eyes achieving 20/25 was higher with DMEK.^9^ Eye banks have therefore started preparing pre-cut and pre-loaded tissues for UT-DSAEK and pre-stripped and pre-loaded tissues for DMEK.^8 10–15^ This had led to a reduction in tissue wastage, better tissue validation and quality control and less surgical time.^16^ In order to overcome the graft preparation issues in theatre, the number of pre-loaded tissues offered by the eye bank has increased rapidly.^4–6^ It has also been shown that UT-DSAEK shows similar results to DMEK in terms of visual acuity, even if there is still a difference in postoperative complications.^2 17^ It is unclear, however, if this can be extrapolated to pre-loaded tissues. The purpose of this study was, therefore, to compare the clinical outcomes and complications of patients treated with pre-loaded UT-DSAEK (pl-UT-DSAEK) and pre-loaded DMEK (pl-DMEK).

## Methods

In this case series, all records from patients treated for endothelial dysfunction due to Fuchs endothelial corneal dystrophy (FECD) and pseudophakic bullous keratopathy (PBK) who had undergone a DSAEK using pl-UT-DSAEK or a DMEK using pl-DMEK between March 2017 and October 2019 were included. Both types of EKs graft were pre-loaded at Fondazione Banca degli Occhi del Veneto (Venice, Italy) and shipped to The Royal Liverpool University Hospital (Liverpool, UK). The retrospective data collection was approved by the institutional review board (A0002786). Exclusion criteria were patients with anterior segment dysgenesis, vitreoretinal disease, previous glaucoma surgery, trauma and a history of uveitis. Surgery was performed by a trainee or consultant for pl-UT-DSAEK and pl-DMEK.^8 10^ Data such as gender, age at the time of surgery, primary diagnosis, donor endothelial cell density (ECD), time from graft preparation in the eye bank to surgery, surgery details (graft diameter, graft thickness and combination with phacoemulsification), best corrected visual acuity (BCVA) and re-bubbling procedure were collected.

Statistical analysis was performed using Stata 14.0 (StataCorp, College Station, Texas) and a p value of less than 0.05 was considered statistically significant. Quantitative variables were tested for normality using the Shapiro-Wilk test. Two sample t-test or Mann-Whitney U test were used according to data distribution. Paired tests were used to compare preoperative and postoperative variables within the same group. Pearson’s χ^2^ test was used to determine whether there was a difference in the number of patients who had a final BCVA 0.2 LogMAR (20/30) and if there was a difference in the rate of re-bubbling.

## Results

Fifty-six patients were included (24 men and 32 women). Thirty-one patients received a pl-UT-DSAEK (69.3±13.0 years) and 25 received a pl-DMEK (77.56±9.7 years; p=0.02). Preoperative BCVA was similar in patients receiving pl-UT-DSAEK (1.09±0.7) and pl-DMEK (0.84±0.58; p=0.17).

There was no significant difference in donor parameters between groups, that is, ECD was 2562±111 cells/mm^2^ and 2560±100 cells/mm^2^ (p=0.99) and time from harvesting to surgery was 2.9±0.7 days and 3.1±0.8 days (p=0.79), respectively, in pl-UT-DSAEK and pl-DMEK groups, respectively. Graft diameters were significantly larger in the pl-UT-DSAEK (9.3±0.25 mm) compared with the pl-DMEK group (8.39±0.27 mm; p<0.01). Graft thickness in pl-UT-DSAEK was 75.29±15.4 µm before transplantation. There was no significant difference in the number of patients with FECD receiving pl-UT-DSAEK (52%, 16/31) and pl-DMEK (72%, 18/25; p=0.17) or in those with PBK receiving pl-UT-DSAEK (48%, 15/31) and pl-DMEK (28%, 7/25; p=0.17).

There was a significant difference in BCVA between the pl-UT-DSAEK group and pl-DMEK group at 12 months (0.37±0.37 vs 0.17±0.20; p<0.01) and in the number of eyes with final BCVA of 0.2 (20/30) or higher in the pl-DMEK group (76%, 19/25) compared with those who received pl-UT-DSAEK (34.4% 13/31; p=0.01).

Patients who had received a pl-UT-DSAEK had significantly fewer re-bubbling procedures compared with those who received a pl-DMEK (12.9% vs 44.0%; p<0.01). Results summarised in table 1.

**Table 1:** Summary of patient, donor details and outcome analysis for non-failed pl-UT-DSAEK and pl-DMEK

	pl-UT-DSAEK	pl-DMEK	P value*
Outcome analysis (*p<0.007)			
Combined cataract surgery	35.5%	60.0%	0.07
Preoperative BCVA (LogMAR)	1.09±0.7	0.84±0.58	0.17
Postoperative BCVA 12 months (LogMAR)	0.37±0.37	0.17±0.20	**<0.01**
Patients with postoperative BCVA ≥20/30	13 (34.4%)	19 (76%)	**<0.01**
Re-bubbling	12.90% (4/31)	44.00% (11/25)	**<0.01**

The column on the right expresses the *p value which was corrected for multiple tests (p=0.05/5=0.01).

BCVA, best corrected visual acuity; DMEK, Descemet membrane endothelial keratoplasty; DSAEK, Descemet stripping automated endothelial keratoplasty; pl-DMEK, pre-loaded DMEK; pl-UT-DSAEK, pre-loaded ultra-thin DSAEK.

Combined cataract surgery occurred in 35.5% (11/31) and 60.0% (15/25) of those patients receiving a pl-UT-DSAEK and a pl-DMEK, respectively (p=0.07). In pl-UT-DSAEK group 3 of 11 patients who had undergone combined surgery had re-bubbling compared with 1 of 20 who had sequential surgery. In the pl-DMEK 8 of 15 with combined surgery had re-bubbling compared with 3 out 10 who had sequential surgery. Although there was no significant difference in the rate of re-bubbling between patients who had combined and sequential surgery within each group, overall (both pl-DSAEK and pl-DMEK) there was a significant increase in the re-bubbling rate in those patients who had combined cataract and EK (both pl-DSAEK and pl-DMEK) surgery (26 and 4 vs 15 and 11, p=0.018).

## Discussion

The results from our study indicate that pl-DMEK is associated with a better BCVA at 12 months after surgery compared with pl-UT-DSAEK and a higher percentage of patients achieved ≥20/30, but at the same time it is also associated with a higher rate of graft detachment requiring re-bubbling.

Our data on visual acuity with eye bank prepared tissues are in agreement with that reported by Chamberlain *et al*,^18^ using surgeon prepared tissues, who reported that patients undergoing DMEK achieved better visual results at 12 months postoperatively compared with UT-DSAEK. In contrast, a more recent study by Dunker *et al*,^9^ also with tissue surgeon prepared tissue found no difference in BCVA at 12 months between DSAEK and DMEK even though there was higher percentage of eyes achieving 20/25 or above in the DMEK group. It must be noted that the retrospective nature of our study without randomisation of patients in the pl-DMEK and pl-UT-DSAEK groups, may represent a selection bias. Even if baseline characteristics showed no major differences between the two groups, it is conceivable that more complex and more advanced cases where allocated to the UT-DSAEK group possibly resulting in lesser chance of visual improvement. Furthermore, a higher percentage of triple procedures (cataract surgery plus EK) in the pl-DMEK group may have been a confounder.

In our cohort we report a higher detachment rate in the pl-DMEK group compared with the pl-UT-DSAEK (44% vs 12.9%). Graft detachment is recognised as the most common complication after EK.^2 19^ The rate has been reported as 34.6% in a multi surgeon setting and 4% in a single case series of DMEK surgeries.^20^ Our re-bubbling rate in the pl-DMEK group was higher. We speculate that exposure of Descemet’s membrane to dextran containing media may result in the deposition of a thin film on the Descemet’s membrane that may interfere with the adhesion of the graft to the stroma. While similar results have been recorded for DSAEK surgery, where graft detachment rates vary widely among studies with figures ranging between 0% and 82% with an average of 15%.^21^ UT-DSAEK has been reported as leading to better visual acuity compared with DSAEK without an increase in the dislocation rate.^22^ In a case series of large pre-loaded UT-DSAEK (9.5 mm), we reported a 23% rate of graft detachment.^8^ In this study, detachment rate of pl-UT-DSAEK was 12.9%.

In a series of 315 eyes Terry *et al*,^23^ reported no significant difference in DSAEK dislocation rates between eyes receiving DSAEK (4%) and triple DSAEK surgery (1.8%; p=0.33) indicating that DSAEK combined with cataract extraction and intraocular lens implant does not increase graft detachment rates. Leon *et al*,^24^ showed more frequent graft detachment and re-bubbling rates when DMEK was combined with cataract surgery, reaching values of 34.10%. In contrast, Chaurasia *et al*,^25^ in a study on 492 patients, re-bubbled only those detachments showing worsening or impairing vision with an overall re-bubbling rate of 29% to 30%. A further study assessing the lens status on DMEK graft detachment did not show any significant difference comparing triple procedure, DMEK only in phakic eyes and DMEK only in pseudophakic eyes with an overall 23.1% re-bubbling rate.^26 27^ Studies on pl-DMEK detachment rate are limited. Busin *et al*, in 2018, showed a 19.6% detachment rate, more often associated with triple procedures while in the same year Newman *et al*,^12^ reported a re-bubbling rate of 14.4%.^10^ In both studies almost all patients had FECD and different preservation conditions in comparison to ours. We found that combined cataract surgery to be a risk factor for re-bubbling, being increased the re-bubbling rate in those patients who had combined cataract and EK. Although we did not find a significant difference between the two groups, the absence of a significant effect in the pl-DMEK may be due to sample size which was only powered to 25.3%.

In conclusion, ready-to-use eye bank prepared tissues has obvious advantages that include decreasing surgical stress and tissue wastage, shortening of operating times and avoiding postponements or cancellations on the day of surgery due to complications in the preparation phase.^7^ Furthermore, eye bank prepared tissues has greater consistency and provides validated tissue in terms of endothelial cell count and graft thickness after preparation.^28–30^ The relatively high rate of graft detachment of pl-DMEK compared with pl-DSAEK is, however, of some concern.
